# Attitudes of Polish Seniors toward the Use of Public Space during the First Wave of the COVID-19 Pandemic

**DOI:** 10.3390/ijerph17238885

**Published:** 2020-11-29

**Authors:** Beata Fabisiak, Anna Jankowska, Robert Kłos

**Affiliations:** 1 Department of Furniture Design, Faculty of Forestry and Wood Technology, Poznan University of Life Sciences, 60-637 Poznań, Poland; robert.klos@up.poznan.pl; 2 Department of Economics and Economic Policy in Agribusiness, Faculty of Economics, Poznan University of Life Sciences, 60-637 Poznań, Poland; anna.jankowska@up.poznan.pl

**Keywords:** furniture, design, public areas, aging population, coronavirus, lockdown

## Abstract

The number of seniors rises worldwide. The lockdown of public institutions caused by COVID-19 influenced the lives of many of them. In the new reality, owners and managers of public spaces need to rethink the way they provide their services, and redesign public spaces to meet the needs of senior citizens. This requires the recognition of the needs of seniors concerning the use of public spaces in the times of the COVID-19 hazard. To investigate this issue, survey studies with 1000 respondents aged 65+ were conducted. The implementation of the obtained data in the process of redesigning public spaces may facilitate the opening up after the lockdown. Taking into account the requirements of a very large group of citizens being seniors is crucial, as it was found that 55% of respondents will also be afraid to use public spaces after the COVID-19 lockdown. The selected ideas that could minimize the feeling of fear when using public spaces after the lockdown were evaluated by seniors.

## 1. Introduction

Population aging and increased urbanization have challenged governments and civic organizations to consider how to develop a community that is accessible for all of its inhabitants in the best possible way [1,2]. Polish people are among the fastest ageing societies in Europe [3]. The analysis of the increase in the share of the population aged 65 years or over between 2009 and 2019 indicates that the mean value for the European Union is 2.9 percentage points, while for Poland, it is 4.2 percentage points. Thus, together with Finland (5.1), Czech Republic (4.7), Malta (4.5), and The Netherlands (4.2) Polish society is aging at a fast pace. Moreover, EUROSTAT predicts that by 2050 the median age in countries such as Malta, Poland, Slovakia, and Cyprus will increase by as much as eight years or more, constituting the highest increase in Europe [4]. The latest forecasts of the Polish Central Statistical Office (GUS) show that people aged 65 and more will comprise 31.5% of the Polish population in 2030 [5]. The rapid demographic change can be observed worldwide [6,7]. To engage and assist cities to become more “age-friendly”, the World Health Organization (WHO) prepared the Global Age-Friendly Cities Guide and a companion “Checklist of Essential Features of Age-Friendly Cities” [8]. The concept of age-friendly public space hides such features as accessibility, intuitiveness and ease of use, safety, and encouraging various types of activities [9]. Among the issues listed in the document that can play the significant role in the current situation are undoubtedly providing public areas that are clean and pleasant or providing green spaces and outdoor-seating furniture that are sufficient in number, well-maintained, and safe [8]. The demographic change undoubtedly has an impact on the social and economic situation of many countries. However, in 2020 another factor arose, developing even in a bigger pace and almost immediately affecting the lives of millions worldwide. The appearance of COVID-19 forced governments and societies to face another big challenge—the lockdown and its implications on senior population and the use of public space. In connection with taking the decisive steps to stop the spread of the SARS-CoV-2 coronavirus, the Polish Government Crisis Management Team with the participation of the Minister of Culture and National Heritage decided to temporarily close cultural institutions, philharmonics, operas, operettas, theaters museums, cinemas, culture centers, libraries, art galleries and schools, universities, and artistic education institutions, starting from 12 March 2020. Moreover, public green areas, particularly parks, promenades, boulevards, botanical, zoological, and historic gardens, were closed in Poland since 1st of April [10,11]. The green areas were reopened on 19th of April; however, for other public institutions, including cultural ones, the reopening process started from 4th of May 2020. The specific date of opening of a given facility was decided by the managing body of a given institution, after the consultation with the district sanitary and epidemiological station. Nevertheless, since that time, much has been said regarding the need to change the behavior patterns in public spaces in order to minimize the risk of infection. Physical distancing has been recommended, requiring people to maintain the distance of 2 m between each other. People were asked to avoid crowded places and social gatherings.

Public spaces play a significant role in the lives of the citizens, as well as in the development of the society. Developing environments responsive to the aspirations and needs of seniors has become a major concern for social and public policy [12]. Wilk [13] highlights that, in order to support the formation of social bonds, the spaces necessary for their creation should be arranged. The user enters the public space immediately after crossing the threshold of his/her household [14] and that has been experienced especially during the lockdown phase. Public space can be an open one (located outdoors) like parks, promenades, boulevards, squares, outdoor gyms, etc., and a closed one (located indoors) like the space in municipality halls, restaurants, cultural institutions (museums, theaters, libraries, etc.), shopping malls, healthcare facilities, etc. Public space can be also defined as a geographical dimension in which there exists free, legal, unbiased access for all [15]. Public spaces have many functions: They allow the users with spaces for relaxation, physical activity, or networking [16]. The longer people stay in the public space the more inspired they are to establish contacts with each other, and this in turns builds the social engagement and the feeling of being the part of the society, which is important for the well-being of senior citizens [17]. If the public space is not adapted to the needs of seniors, it becomes for them an environment full of obstacles and barriers [18]. However nowadays, due to COVID-19 pandemic, it may also evoke psychological fear related to the risk of infection. Up till now, the fears of seniors concerning the use the public space were mostly connected with the crime issues [19,20] or the fear of outdoor falls [21], focusing more on the issues of environmental obstacles and adjustment of the public space to the physical abilities of seniors [22,23,24].

Public spaces are of key importance for sustaining the public realm. This is especially significant now, as societies of the modern world are no longer dependent on town squares or piazzas for basic needs; therefore, well-designed urban spaces are required for the social and psychological health of modern communities [25]. Consequently, great interest is currently being paid in regard to making public spaces more accessible, safe, and comfortable to as many citizens as possible. Most of the studies related to this aspect have concentrated on the recognition of seniors’ needs regarding outdoor public spaces, such as green areas, e.g., References [26,27,28,29,30], or they have focused on street furniture and outdoor urban spaces in general, e.g., References [31,32,33,34,35]. Earlier, the survey research aimed at recognizing the needs of seniors concerning the public space was conducted, for instance, by Turel et al. [36], Risser et al. [37], Francis et al. [38], Mehran and Farkhondeh [39], and Michael et al. [40]. Mitchell and Burton [41] focused on interviewing seniors in the mild or moderate stages of dementia and the ones without dementia. Despite all of those valuable studies, the conditions of using public spaces have changed significantly over the spring of 2020.

The COVID-19 pandemic has caused tremendous changes in the lives of many societies in the world. An interesting study of Ugolini et al. [42] is dedicated to the effects of the COVID-19 pandemic on the use and perceptions of green space in six European countries. It, however, does not focus on the perceptions of senior citizens. Lebrasseur et al. [43] pay attention to the impact the pandemic has had on people with physical disabilities, proving more attention and research is needed to focus on the needs of this group of citizens in this challenging period of our history. Moreover, Goggin and Ellis [44] notice the needs of people with disabilities during the pandemic. Nevertheless, not many studies concerning the impact of COVID-19 on the lives of populations focus on seniors. It is notable to mention here the works of Suzuki et al. [45], Garcia-Fernanez et al. [46], Armitage and Nellums [47], and Lee et al. [48]. The pandemic affected seniors in many various dimensions: in a direct way, due to the risk of infection and death, as well as in the indirect way, due to the lockdown that increased the feeling of loneliness and isolation by not being able to use the public space as freely as before the pandemic.

**Figure 1:**
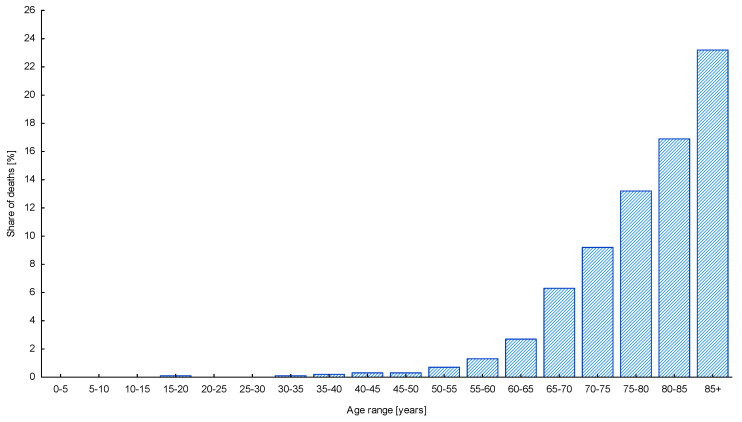
Distribution of deaths due to COVID-19, in Poland, with regard to the age group. Source: Chancellery of the Polish Prime Minister, press conference, 5.11.2020 (data period 4.03–4.11) [51].

The research of Polish Central Statistical Office [5] that was conducted prior to the pandemic, and was attended by 13.3 thousand people over 65 years of age, showed that as many as 67% of them used to spend their free time outdoors, e.g., walking and spending time outside the household, at least once a month. Nevertheless, the medical experts say that it is the oldest population that is most at risk of infection of COVID-19 [49,50]; thus, many seniors made the decision to self-isolate. In many European countries, including Poland, the governments advised seniors to stay at homes, in order to protect this vulnerable social group. The high risk the infection of COVID-19 may bring to the older citizens is confirmed by the data on the mortality due to COVID-19 with the regard to the age group (Figure 1).

As the pandemic slows down and public spaces start to be reopened, their owners also need to rethink the way they operate to allow for the safest possible usage of their premises. It is essential to be prepared for the changes and modify the range of offered services accordingly. Thus, it is necessary to study the changes in the attitudes of seniors toward the use of public spaces caused by the appearance of the COVID-19 pandemic.

The main purpose of this article is to show the scale of the problem concerning the use of public spaces by seniors in the times of the first wave of the COVID-19 pandemic and to present their attitudes toward public space that were affected by the lockdown. The main focus is placed on the recognition of their feeling of safety when using public spaces. Being able to use public spaces supports the willingness to be a visible part of the public life, which naturally affects the well-being of older citizens. The investigated data can be useful for making policy recommendations related to land-use planning, to assist in senior-friendly developments and neighborhood improvements, and to design effective senior health interventions with an emphasis on neighborhood design [41]. Therefore, the obtained results have foremost the valuable implications for designers, managers, and owners of public spaces who quickly need to adapt to new conditions, safety requirements but also customer requests to make their premises safe and attractive again.

## 2. Materials and Methods

### 2.1. Study Design and Setting

In order to acquire the data on the attitudes of seniors regarding the usage of public space during the first wave of COVID-19 pandemic, survey research was developed. The works performed consisted as part of the international study being developed to indicate paths for potential areas of interests when solving the problems seniors face while functioning in the public space. The questionnaire was developed by experts, representing various fields, such as wood technology, design, geriatrics, and robotics from nine countries located in the Baltic Sea region. When the pandemic hit, the research consortium decided to expand the scope of the issues investigated and recognize also the citizens’ perception toward the use of public space during the first wave of COVID-19 pandemic. The survey format consisted of open- and closed-ended dealing with a wide range of problems seniors face while using the public space. The results presented below refer to the questions concerning the needs and fears accompanying older people in the view of the changes in social life caused by the presence of the COVID-19 virus. The focus was on the subject of outdoor public space arrangement, design elements, furniture, and problems arising from the current situation. The study was performed at the turn of April and May of 2020.

### 2.2. Participants and Survey Procedures

The research was conducted in the form of electronic survey that reached 1150 people aged 65+ living in Poland via a professional online survey platform (4P, Warsaw, Poland). The method used was a Computer-Assisted Web Interview (CAWI). The survey was programmed in CADAS software 5.00 (Warsaw, Poland). Due to the lockdown phase and the high risk of infection, especially among the senior population, it was decided to perform the study via a remote method. The distribution via a platform did not allow for personal identification of individual respondents.

Taking into account the percentage of completed surveys, a statistical analysis was conducted on the data obtained from 1000 seniors. The sample population consisted of 57% men and 43% women (Table 1). The researched population constituted of seniors living both in the urban and rural areas of Poland.

### 2.3. Analysis Procedures

The questions analyzed in this work were closed-ended questions, mostly of multiple choice, with the open-answer possibility in each question. The answers provided under the open-answer option were analyzed separately. If various respondents indicated similar answers, the new codes were assigned to those responses, and that allowed for further comparative analysis of this data. The open-ended answers constituted a significant part of the study, as they enabled the respondents to describe, in more detail, their personal observations or provide comments about their doubts, worries, or possible solutions that could be incorporated in the design and development of public spaces.

The gathered data were coded and subjected to statistical analysis. The coding was done by transferring each item of the questionnaire into a variable reflecting the answer of the respondent. Using the statistical grouping method, we determined the characteristics of the needs and attitudes of seniors related to the analyzed subject. The analysis was conducted by using STATISTICA 13 PL (Dell, Round Rock, TX, USA).

As the life of many of us has changed due to the COVID-19 pandemic, and as the senior population was mainly hit by the limitations posed by the governments, in order to protect the citizens from spreading of the coronavirus, we decided to investigate whether the fears of using the public space be vivid also after the lockdown, what were the issues respondents missed the most during the first wave of the lockdown, and what could be done to minimize the insecurity while using the public space in the times of COVID-19 pandemic.

## 3. Results

The pandemic situation has caused many fears, including those connected with the use of public space. Thus, the first issue we wanted to investigate was whether those feelings will have an impact on the fear of using public spaces also in the time after the lockdown. It turned out that only 15% of seniors state they will be afraid to use the public space after the lockdown; however, it is important to notice that almost 40% declared they will be “a little” afraid. That gives an image of the population where 55% will be at least a little afraid to use public spaces.

**Figure 2:**
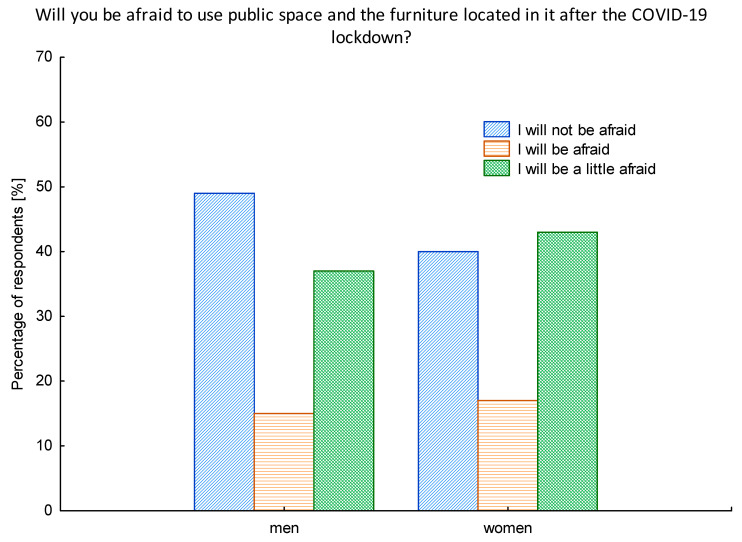
The structure of the investigated population in relation to the feeling of fear for using the public space after the lockdown in relation to gender. Source: Authors’ own elaboration based on the performed survey research.

We performed a more comprehensive analysis of this issue by taking into consideration the gender factor. It turned out that the feeling of fear when using the public space was more frequent among women population (60%) (Figure 2).

Furthermore, we were wondering whether the fear is connected with the place of living. We assumed that seniors living in urban areas, in more populated regions will be more afraid of using the public space due to the COVID-19 risk of infection. However, it turned out that the appearance of those emotions was not connected with the type of area of living. Both in urban and rural areas, a large number of seniors was feeling at least a little afraid to be using the public space after the lockdown (55% and 54%, respectively).

**Figure 3:**
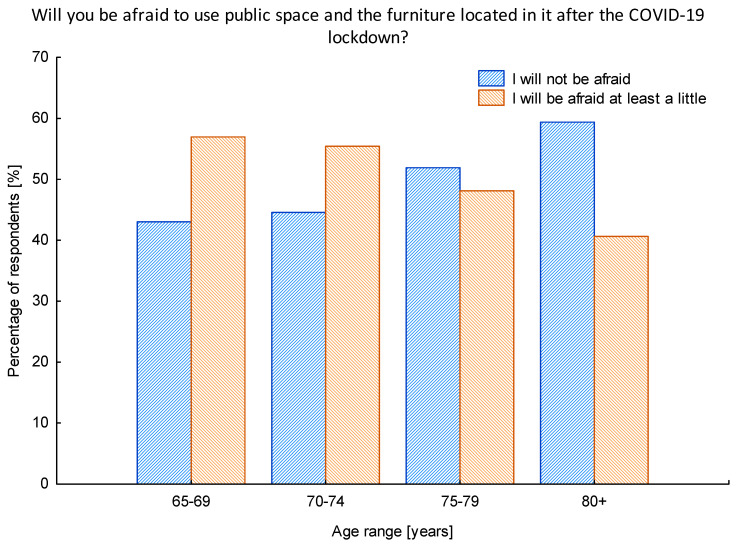
The structure of the investigated population in relation to the feeling of fear for using the public space after the lockdown in relation to age. Source: Authors’ own elaboration based on the performed survey research.

An interesting result was obtained from the analysis of the attitudes toward using of the public space in relation to the age of respondents. According to the medical data (Figure 1), the higher the age, the bigger the possibility of having a severe course of the disease, including the death of the patient. Thus, we expected a strong positive correlation between the age of respondents and the number of people experiencing the fear of using the public space after the lockdown. It turned out, however, that the correlation between those variables was a negative one, meaning that the older the people were, the lower the number of them who were afraid of using the public space (Figure 3). Seniors’ fear of using public space and the furniture located in it decreased with the age of seniors. In this case, the relationship between the age of seniors and the percentage of people who are afraid to use public spaces can be described by the linear function (y = −1.240 × x − 142.03) with a high correlation coefficient (r = −0.944).

**Figure 4:**
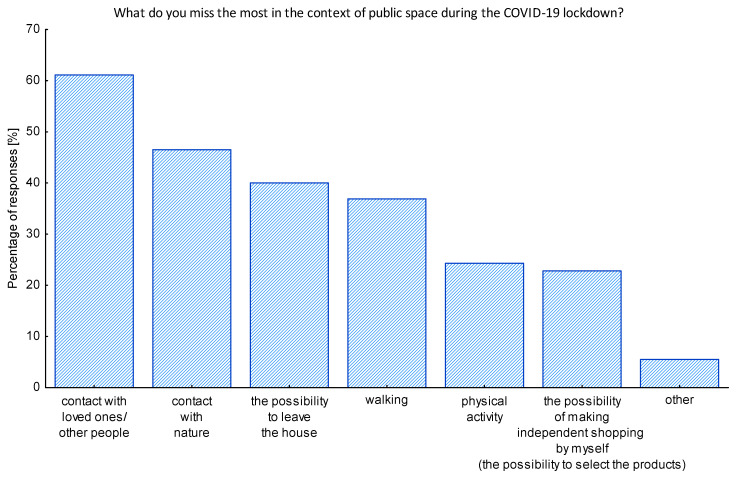
The issues lacked the most during the pandemic lockdown. Source: Authors’ own elaboration based on the performed survey research.

While investigating the feelings of missing something during the lockdown situation, it turned out that, for over 60% of the surveyed seniors, the most severe was the lack of the direct contact with the close ones and other people (Figure 4). Almost half of the respondents indicated also that they lacked contact with nature. When the open-ended question appeared to indicate other issues seniors lack the most in the pandemic period, some of them replied they miss their additional professional activity, contact with the doctors, and the possibility of traveling. Only 1.5% admitted they do not miss any of the listed issues during the pandemic. It is however very important to notice that all respondents stating the restrictions did not affect their lives so much were all living in rural areas.

Furthermore, it is also worth highlighting that 90% of respondents prefer to use open public spaces (like parks, promenades, boulevards, squares, etc.) in contrary to the closed ones (located inside buildings like municipality halls, shopping malls, offices, etc.). This is essential information for the “after lockdown” phase where some of the services will be allowed to be reopened only in the outdoor space.

**Figure 5:**
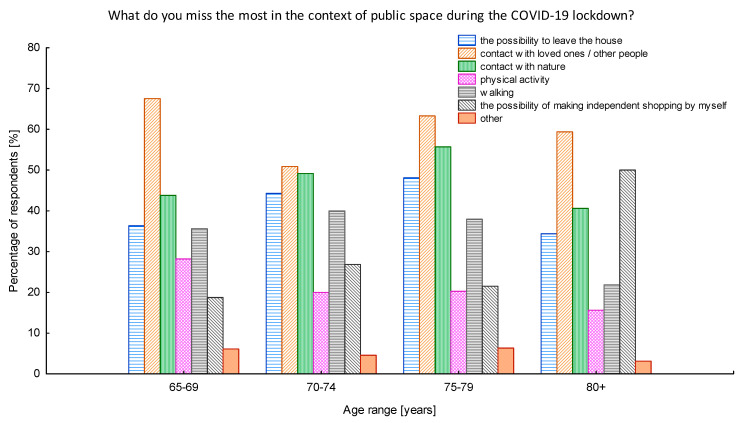
The issues lacked the most during the pandemic lockdown with regard to the age of respondents. Source: Authors’ own elaboration based on the performed survey research.

The relationship between the age of seniors and the issues they lacked the most during the first wave of COVID-19 pandemic is shown in Figure 5. The obtained results show that, with the increase in the age of respondents, the number of people who lacked the most the opportunities to make independent purchases raises. This relationship can be described by a linear function (y = 1.833 × x − 106.39) with a correlation coefficient (r = 0.737).

In turn, the older the seniors were, the less they felt the lack of physical activity and the possibility of going for a walk. These correlations were also described with linear functions (y = −0.859 × x + 97.43 for walking and y = −0.846 × x + 83.61 for physical activity) having correlation coefficients of r = −0.599 and r = −0.923, respectively.

**Figure 6:**
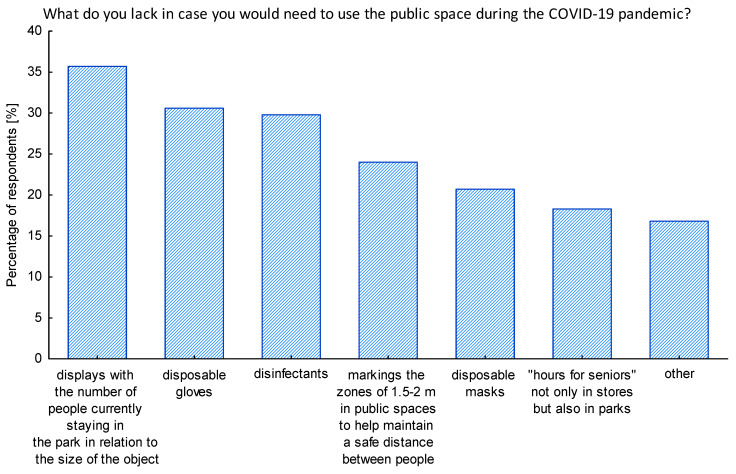
The things that according to seniors would be needed the most in the public space during pandemic. Source: Authors’ own elaboration based on the performed survey research.

Even during the pandemic lockdown, some seniors needed to leave their homes. We investigated what were the things they lacked the most in public spaces in that situation. It is interesting to note that over 35% lacked the information on how crowded the place they wanted to visit is. Thus, they liked the most the idea of seeing screens indicating the number of people currently being present in a park, with reference to the area of the park, in order to feel more safe and make a decision to enter (Figure 6). Respondents indicated also they would like to see disposable gloves and disinfectants being available in public spaces. Among the open answers to this question, respondents mentioned safe public transport with smaller number of users, the general feeling of safety in the public space, adherence to the safety COVID-19 rules by other citizens, and safe and senior-friendly pharmacies. Others pointed out that they try to avoid the public space during the lockdown and use it only when it is necessary.

**Figure 7:**
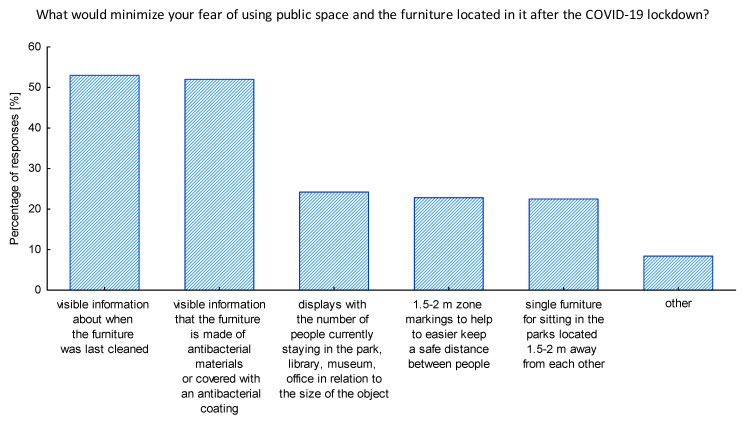
The ideas that could minimize the feeling of fear when using the public space after the lockdown. Source: Authors’ own elaboration based on the performed survey research.

Furthermore, to be better prepared for the future actions we studied the opinions of seniors toward the potential solutions that could be implemented in public spaces to minimize their fear of using them. It turned out that over half of the respondents would like to see in the public space the information that furniture is made of antibacterial materials or covered with an antibacterial coating, also they were willing to see a visible information concerning when the furniture was last cleaned (Figure 7). It is important to note that, even though the antibacterial solutions are not suitable to combat COVID-19, they can bring in a fair portion of security to the perception of the users of the public space. The majority of respondents choosing the open-answer possibility listed the cleanliness as a general factor enhancing their security when using the public space in pandemic times. One person wished for mobile light covers that could be changed and washed.

**Figure 8:**
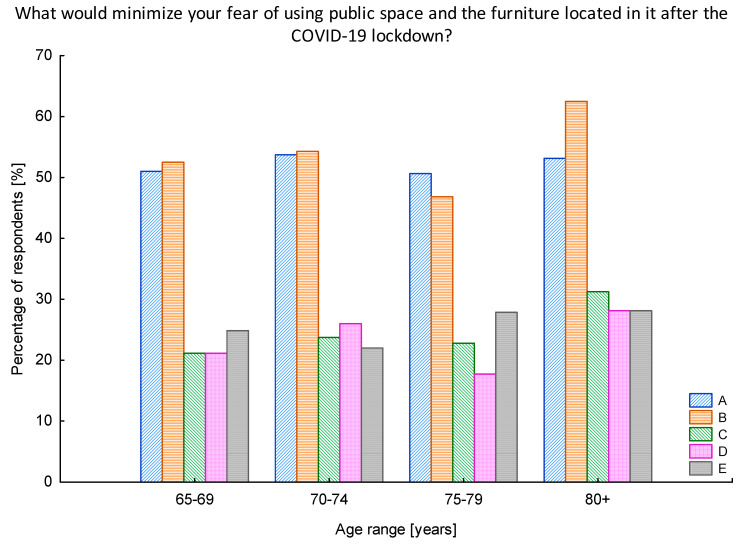
The ideas that could minimize the feeling of fear when using the public space after the lockdown, with regard to the age of respondents. A—visible information that the furniture is made of antibacterial materials or covered with an antibacterial coating; B—visible information about when the furniture was last cleaned; C—single furniture for sitting in the parks located 1.5–2 m away from each other; D—1.5–2 m zone markings to help to make it easier to keep a safe distance between people; E—displays with the number of people currently staying in the park, library, museum, and office, in relation to the size of the object Source: Authors’ own elaboration based on the performed survey research.

Furthermore, we decided to investigate whether the preferred solutions were connected with the age of respondents (Figure 8). Nevertheless, no significant correlations were found. It turned out that the care for the cleanliness of the furniture located in the public space was of paramount importance constituting the leading need of users recognized in all analyzed age groups (ranging from 47% of respondents in the age group of 75–79 up till 63% in the group of over 80 years old).

**Figure 9:**
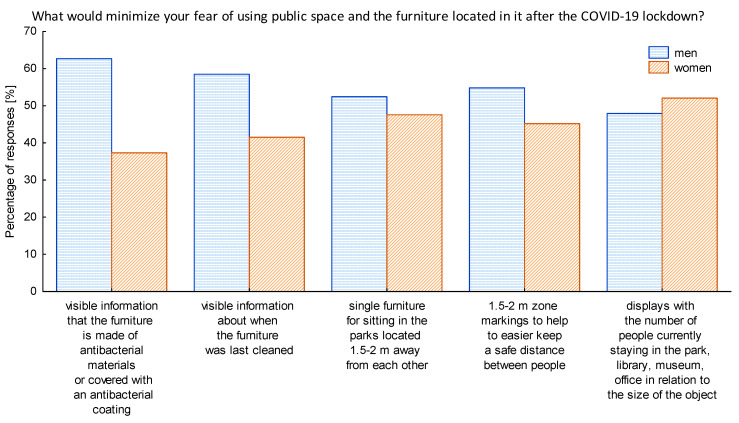
The ideas that could minimize the feeling of fear when using the public space after the lockdown most liked by the respondents with regard to gender of the respondents. Source: Authors’ own elaboration based on the performed survey research.

Taking into consideration the gender factor indicated that over 60% of men would like to see the information that the furniture in public spaces is made of antibacterial materials or is covered with an antibacterial coating, while 59% would like to see visible information about the timetable of the cleaning activities in a given space (Figure 9). Women, in turn, were the most enthusiastic about the idea of screens indicating the number of people currently using the park, the library, the museum, or the office, etc. in relation to the size of the object, as well as a new designs of furniture for sitting for one person located in the parks with the distance of 1.5–2 m from each other, to facilitate the physical but not social distancing.

## 4. Discussion

The idea of the study arose together with the first wave of COVID-19 lockdown that took place in Poland in spring of 2020. Many administrative decisions limited the public life and the ability to use public spaces (both open and closed ones), including parks, boulevards, cultural institutions, etc. Moreover, after the slow re-opening in May of 2020, the rules of using the public space have been changed, implementing physical distance and avoiding the crowds. Real-time data published by the World Health Organization [50], as well as the Polish Ministry of Health [51], showed that the risk of COVID-19 infection is especially dangerous for the senior population, and thus many seniors made decisions about self-isolation. During the lockdown phase, the representatives of the Polish government also advised seniors to stay at home. It resulted in a number of consequences that hit the senior population, especially those living alone. For them, public spaces provide social interactions that mitigate isolation and loneliness [15]. Moreover, results of our research confirmed that over half of respondents missed the contact with the loved ones, with other people, and with nature the most during the time of the lockdown. This pays attention to the role of green public spaces of various types, from urban parks to pocket gardens, with the overall goal of providing a wide range of opportunities to access and enjoy the nature in the city.

A surprising result was connected with the overall feeling of fear to use the public space also after the pandemic peak. We were wondering whether the uncertainty encountered during the lockdown will remain with seniors for a longer time. Unexpectedly, the older the respondents were, the fewer of them that were feeling anxiety to use the public space in the pandemic times. Similar results were obtained by Garcia-Fernandez et al. [46] on a sample of Spanish seniors. As far as mental health was considered, it turned out seniors were less vulnerable than younger participants of the study. We do agree with the possible hypothesis stated also by Garcia-Fernandez et al. [46] that the senior population is a generation that has a greater resilience than the younger. We can clearly observe various generations that are represented in the sample population of our research, as there are representatives of the World War II/interwar period generation and the baby boomer generation. The World War II/interwar generation is used to harsh living conditions; this is a modest generation that does not require too much from external environments and tries to not cause problems to others. Individuals of this generation are loyal and disciplined [52,53]. This is also reflected in the results obtained. Even though this group of users is supposed to feel the biggest fear due to the risk of COVID-19 infection, more representatives of the baby boomer generation state they will be at least a little afraid of using the public space after the lockdown. The difference in senior generations is seen also in the results describing the issues seniors lacked the most during the first wave of COVID-19. Taking into consideration that the age factor revealed that seniors aged 80+, apart from lacking the contact with other people and nature, were mostly worried about the limitation in the possibilities of making independent shopping. This demonstrated how crucial it was for them to remain independent and being able to cope with the everyday shopping activities.

After the first wave of the pandemic, scientists, designers, entrepreneurs, and governments, as well as society, had a clear vision that the world we know is going to evolve and change due to the pandemic experiences. Many already published some of their thoughts on how the pandemic will change the relationship with public space [15,54,55,56]. We also wanted to contribute to the worldwide fight and bring in new insights on the attitudes of seniors in this time of the crisis. From the practical point of view, it was also crucial to recognize the potential solutions that could enhance minimizing those fears bringing back the safety of use to the public space, especially for seniors. Honey-Roses et al. [15] point out that there is a need to re-think urban planning to respond the new behaviors and needs that have arisen from the COVID-19 pandemic. The achieved results demonstrate that over half of the surveyed population will be at least a little afraid to use public spaces. Being aware of that situation can allow also for making actions to combat those fears and minimize the risk of infection due to responsible design of the outdoor and indoor environment.

Once the first wave of COVID-19 was gone public institutions started to be re-opened. Nevertheless, the pandemic was still there not allowing for the full return to the previous lifestyle of societies. Thus managers and owners of public spaces needed to adapt quickly to the new conditions and safety requirements, but also to the new customer requests to make their premises safe and attractive again. The world of design has shown its support, and a range of new ideas has appeared. The initiative of The Design Vanguard named Design for COVID-19 is an inspiring example [57]. Moreover, our research has contributed to the recognition of the first emotions and reactions arose due to the first wave of COVID-19 pandemic allowing for the diagnosis of seniors’ preferences. During our research we gathered the opinions of seniors concerning the potential solutions that could be implemented in the public space to raise the level of safety in the times of COVID-19 and thus minimize the fear of using them (Figure 7, Figure 8 and Figure 9). What we have noticed was a raising interest in the hygienic issues and even though the antibacterial materials and covering the furniture with an antibacterial coating will not help to stop the COVID-19 virus, respondents had a feeling that providing any kind of solution for enhancing the hygienic values would make them feel more safe in the public space. This is in line with the approach to include health considerations in the creation of public spaces. Honey-Roses et al. [15] highlight that although this theme is not new, and numerous authors have dedicated their work to support the design of public space with the health issues as a bottom-line [58,59], it has never before been such crucial and such widely discussed and accepted. Moreover, experts worldwide strive to face the challenge of the new COVID-19 pandemic. French material manufacturing company Serge Ferrari Group has developed and patented a coating technology for its membranes which makes its surfaces virucidal [60]. This solution, due to the use of the technology based on silver particles, is capable of eliminating coronaviruses by up to 99.5%. Thus, the company managed to find a way to prevent fabrics from becoming propagation sources for viruses and bacteria. The solution can be applied on handles, railings, furniture, etc. We would like to pay attention to the fact that half of the seniors participating in the research admitted that providing visible information when the furniture or other elements of the public space were cleaned is also a good way to minimize their fear of using the public space in the times of pandemic. Furthermore, respondents pointed out that the cleanness of the furniture located in the public space, even before the pandemic hit, was one of the major issues discouraging them to use the public space.

It is also worth noting that the solution of marking the zones of 1.5–2 m to facilitate keeping physical distance was chosen as preferable by only 23% of the respondents. It is surprising as those solutions were applied successfully in a number of cities worldwide, e.g., in Toronto, New York, and Melbourne [61]. Moreover, in Poland, we had an interesting example of such activity in Elbląg, where the grass was cut in a way to leave a chessboard pattern of physically distancing squares [62].

The presented results show seniors’ first emotions when using the public space in the time of the first wave of COVID-19. Further research is planned as the second wave of pandemic is already much stronger than the one that hit during the spring time. Furthermore, additional factors have arisen and as a long-term tiredness and months of living in a pandemic risk might have further implications on the final results and relationship to the use of public space. Nevertheless, the experiences gained from the first wave can be valuable sources of inspiration and facilitate better preparation for the re-opening processes in the future.

As far as the limitations of the study are concerned, it needs to be stated that, due to the realization of the study in the most dangerous time of pandemic peak, we decided to perform the survey research fully by using the remote method of Computer-Assisted Web Interview. That could exclude the part of digital non-users. Nevertheless, the high response rate to the survey and the rising number of Internet users in Poland that, in September of 2020, accounted for 73% of Polish population [63], reassured us that the actions taken were justified. Another limitation might be the disproportional number of participants in different types of demographics that may induce bias and affect the final results. This is the result of the decision to conduct the research in the electronic form through an unrestricted self-selected survey. Therefore, in the future, we plan an update of the research to confirm and expand the results, showing the situation after the second wave of the COVID-19 pandemic.

## 5. Conclusions

The research reports on the first moods regarding the use of public spaces seniors have had due to the first pandemic lockdown. It sheds light on the needs and feelings that people aged 65+ have encountered in the spring of 2020. The last part has the biggest potential, as it indicates the evaluation of the selected ideas concerning minimizing the fear of using public space and gives a good inspirational background for the future developments. The pandemic time revealed and strengthen the existing inequalities in the access to the public space [43,64]. Thus, it was crucial for us to dedicate this research to one of the vulnerable target groups, namely seniors. The recommendations enclosed in the “Checklist of Essential Features of Age-Friendly Cities” [8] we mentioned earlier have received a new meaning in the times of pandemic and gained even more relevance. Providing public areas that are clean and pleasant is a clear response to the needs of seniors foremost in the times of COVID-19. The obtained research results show that great attention of scientific institutions and companies should be now paid to the material testing that can provide not only antibacterial but also antiviral surface properties and their practical use in furniture to be implemented in public spaces. Moreover, the next feature of senior-friendly cities reports that it is essential to provide green spaces and outdoor seating that are sufficient in number, well-maintained, and safe. The first wave lockdown situation has showed how relevant were both: the accessible green spaces and the furniture located in the spaces. The great uncertainty on how the relationship toward the public space will be shaped in the near future from one side evokes the need for further, more in-depth analysis, and on the other opens the door for the radical and beneficial changes in the design of the public space. The changes might have even been on the agenda but have never had the sufficient priority to be implemented. Now the time has come to really include the health-based criteria to our thinking of the future of the public space. Furthermore, although much is still to be investigated in this uncertain time, our research shows that the upcoming form and functionality of public spaces will be shaped by the design solutions that will help to create the environment in a manner to secure the maximal safety level but at the same time will allow for the use of public spaces and facilitate interactions between the users of those spaces, as physical distancing does not mean the need for social distancing.

## Figures and Tables

**Table 1:** Demographic profile of the respondents.

Overall Sample (*n* = 1000)			
**Age Range (Years)**		**Living Arrangement**	
65–69	539 (53.9%)	Single households	219 (21.9%)
		Living with spouse (partner)	640 (64.0%)
70–74	350 (35%)	Living with family, children, grandchildren	139 (13.9%)
75–79	79 (7.9%)		
80+	32 (3.2%)	Other	2 (0.2%)
**Gender**		**Type of Household**	
Female	433 (43.3%)	Apartment in an urban area	618 (61.8%)
Male	567 (56.7%)	Apartment in a rural area	60 (6.0%)
		House in an urban area	183 (18.3%)
		House in a rural area	139 (13.9%)

Source: Authors’ own elaboration based on the performed survey research.
